# Clinical implications in the use of the PBC algorithm versus the AAA by comparison of different NTCP models/parameters

**DOI:** 10.1186/1748-717X-8-164

**Published:** 2013-07-04

**Authors:** Antonella Bufacchi, Barbara Nardiello, Roberto Capparella, Luisa Begnozzi

**Affiliations:** 1 Medical Physics, PioXI Clinic and U.O.C. Medical Physics, S. Giovanni Calibita Fatebenefratelli Hospital, Rome, Italy; 2 UPMC San Pietro FBF Advanced Radiotherapy Center, Rome, Italy; 3 U.O.C. Medical Physics, S. Giovanni Calibita Fatebenefratelli Hospital, Rome, Italy

**Keywords:** Dose volume histogram, Normal tissue complication probability, Pencil beam convolution algorithm, Anisotropic analytical algorithm

## Abstract

**Purpose:**

Retrospective analysis of 3D clinical treatment plans to investigate qualitative, possible, clinical consequences of the use of PBC versus AAA.

**Methods:**

The 3D dose distributions of 80 treatment plans at four different tumour sites, produced using PBC algorithm, were recalculated using AAA and the same number of monitor units provided by PBC and clinically delivered to each patient; the consequences of the difference on the dose-effect relations for normal tissue injury were studied by comparing different NTCP model/parameters extracted from a review of published studies. In this study the AAA dose calculation is considered as benchmark data. The paired Student t-test was used for statistical comparison of all results obtained from the use of the two algorithms.

**Results:**

In the prostate plans, the AAA predicted lower NTCP value (NTCP_AAA_) for the risk of late rectal bleeding for each of the seven combinations of NTCP parameters, the maximum mean decrease was 2.2%. In the head-and-neck treatments, each combination of parameters used for the risk of xerostemia from irradiation of the parotid glands involved lower NTCP_AAA_, that varied from 12.8% (sd=3.0%) to 57.5% (sd=4.0%), while when the PBC algorithm was used the NTCP_PBC_’s ranging was from 15.2% (sd=2.7%) to 63.8% (sd=3.8%), according the combination of parameters used; the differences were statistically significant. Also NTCP_AAA_ regarding the risk of radiation pneumonitis in the lung treatments was found to be lower than NTCP_PBC_ for each of the eight sets of NTCP parameters; the maximum mean decrease was 4.5%. A mean increase of 4.3% was found when the NTCP_AAA_ was calculated by the parameters evaluated from dose distribution calculated by a convolution-superposition (CS) algorithm. A markedly different pattern was observed for the risk relating to the development of pneumonitis following breast treatments: the AAA predicted higher NTCP value. The mean NTCP_AAA_ varied from 0.2% (sd = 0.1%) to 2.1% (sd = 0.3%), while the mean NTCP_PBC_ varied from 0.1% (sd = 0.0%) to 1.8% (sd = 0.2%) depending on the chosen parameters set.

**Conclusions:**

When the original PBC treatment plans were recalculated using AAA with the same number of monitor units provided by PBC, the NTCP_AAA_ was lower than the NTCP_PBC_, except for the breast treatments. The NTCP is strongly affected by the wide-ranging values of radiobiological parameters.

## Background

As a result of the increased sophistication of treatment techniques and delivery methods, the accuracy of highly conformal radiotherapy has improved rapidly with technological advances in recent years. However more demanding modern treatment techniques require better modeling of treatment beams and more sophisticated modeling in the presence of inhomogeneities in order to guarantee accuracy in the calculation of dose distribution. In the clinical routine, calculations of dose to the tumor are performed by commercial treatment planning systems (TPS) and the majority of these systems include dose calculation algorithms with a limited ability to account for the effects of electron transport [1]. The Pencil Beam Convolution (PBC) algorithm is commonly used in clinical practice. However it is well known that it has shortcomings regarding the presence of inhomogeneities, particularly in those regions where charged particle equilibrium does not exist [2-5]. The introduction of convolution-superposition (CS) algorithms that better account for electron transport, have enabled improved calculation of dose distribution, principally in the absence of electronic equilibrium [6-11]. In the Eclipse TPS (Varian Medical Systems) the Anisotropic Analytical Algorithm (AAA) is implemented; it is a 3D pencil-beam kernel-based superposition algorithm [12]. The AAA includes separately modeled contributions from three sources: primary photons, extra-focal photons and contaminating electrons; each of these has an associated fluence, an energy deposition density function and a scatter kernel. A better consideration of inhomogeneities is obtained when the AAA is used. The higher accuracy of the AAA, compared to the Eclipse’s Pencil Beam Convolution (PBC) algorithm is well-established [13-16]. Monte Carlo (MC) simulation is considered to be a gold standard in dose calculation, and it is therefore used to evaluate other dose calculation algorithms. Sterpin et al. [17] investigated the accuracy of the AAA in two studies. First the AAA was compared both with MC and measurements in an inhomogeneous phantom. Second, the AAA and MC were compared with four Intensity Modulated Radiation Therapy (IMRT) treatment plans in the presence of inhomogeneous tissue. They showed good agreement between the AAA and MC and evaluated the improved accuracy of the AAA compared to the PBC algorithm.

Studies Fogliata et al. [18] carried out, show how Collapsed Cone (CC) and AAA manifested a high degree of consistency compared to the MC method, when the impact of photon dose calculation algorithms on expected dose distribution in lungs under different respiratory phases was investigated. PBC proved to be severely defective in calculations, particularly for cases where specific respiratory phases (e.g. deep inspiration breath hold) were assumed for treatment.

In the study of Bragg et al. [19] compared to the PBC algorithm, the AAA was not found to significantly alter the quality of IMRT treatments plans for prostate, parotid or nasopharynx. While its more accurate modeling of lateral electron transport demonstrates significant increases in the volume of PTV being underdosed in IMRT non-small cell lung cancer (NSCLC) treatments plans.

Nielsen et al. [20] investigated the differences in calculated dose distributions and NTCP values between six different dose calculation algorithms for NSCLC treatments. The study showed how the calculated NTCP values for pneumonitis were more sensitive to the choice of algorithm than mean lung dose and V_20_ which are commonly used for plan evaluation. Furthermore, NTCP for the lungs was calculated using two different model parameter sets; within each dose calculation algorithm, large differences were found between the calculated NTCP values.

The aim of our study was the retrospective analysis of 3D clinical treatment plans to investigate qualitative, possible clinical consequences of the use of PBC versus AAA, which was considered as benchmark data. The 3D dose distributions of 80 treatment plans at four different tumor sites, produced using PBC algorithm, were recalculated using AAA and the same number of monitor units provided by PBC and clinically delivered to each patient. Similarly to the study of Nielsen et al., in addition to the information relating to the normal tissue/target dosimetry and the Tumor Control Probability (TCP), the comparison was performed investigating the consequences on the dose-effect relations for normal tissue injury comparing different Normal Tissue Complication Probability (NTCP) model parameters extracted from a review of published studies.

Several authors have proposed studies to compute NTCP from a survey on clinical tolerance data [21,22]. After the first parameterization of dose-volume effects reported by Burman et al. [23] and based on the experience from the 2D radiotherapy era, the availability of 3D dose-volume information strongly increased the amount of quantitative data. Consequently much effort was dedicated both to the proposal of reliable dose-volume constraints that demonstrated capability to reduce toxicity, and the development of normal tissue complication probability (NTCP) models properly fitting clinical data. However NTCP models have been re-evaluated using 3D dose data calculated by rather simple computation algorithms; as it is difficult to quantify the clinical consequence of approximate dose calculations, the value of the reported NTCP parameters remains questionable. De Jaeger et al. [24] compared the results of dose calculations in lung tissue using an approximate dose calculation algorithm (the equivalent-pathlength model, EPL) with calculations for a convolution-superposition (CS) algorithm, and the consequences with respect to the estimation of normal lung tissue injury in a group of patients with NSCLC was evaluated [25]. The study demonstrated that when more accurate dose data is available, a re-evaluation of NTCP model parameters is necessary to avoid NTCP being grossly over or underestimated.

The degree to which the NTCP is strongly affected by the wide-ranging values of radiobiological parameters is shown in this study. At present, the evaluation of the optimal values of the radiobiological parameters is difficult; absolute NTCP values are not reliable enough to be considered for evaluating a treatment plan. However the NTCP values have the attractive feature of synthesizing in only one value the whole dose distribution throughout the organ of interest and along with the dose-volume parameters are useful tools for comparing rival plans or for defining dose escalation strategies.

## Methods

### Patient data, treatment planning and delivery technique

3D clinical treatment plans of 80 patients were reviewed for this study. 20/80 were irradiated for left breast cancer with two tangential fields, 20/80 were treated for non-small cell lung cancer (NSCLC) by three-dimensional conformal radition therapy (3DCRT); as the PTV locations varied widely, the beam angles were adjusted for each individual patient to meet both the set dose-volume constraints for Organs At Risk (OAR) and acceptable dose distributions. The tumor was situated in the middle or lower lobe, eight plans required four fields; all others used five fields. 20/80 patients were irradiated with intensity-modulated radiotherapy for head-and-neck cancer using seven coplanar fields arrangement [26]; finally 20/80 prostate cancer 3DCRT treatments plans (five coplanar field technique 0°,45°,90°,270°,315°) were re-evaluated.

The target volumes were defined in accordance with the 1993 International Commission on Radiation Units and Measurements Report 50 (ICRU Report 50). The gross tumor volumes (GTV) included all known gross disease as determined by imaging and clinical findings. GTVs were expanded to yield corresponding clinical target volumes (CTVs) according to clinical assessment in each case.

For breast cancer, the CTV was glandular breast tissue and the PTV was generated by expanding the CTV by 0.7 cm isotropically, except in the direction of the skin surface. For lung cancer, the stage was IIIA/IIIB (T3) with a broad scope of disease; the GTV was similar to the CTV. The radiation oncologist identified the GTV, and using a margin of 0.3 cm, the CTV was delineated. A margin of 0.7 cm for middle lobe tumors and 1.0 cm for tumors in the lower lobes was added to create the PTV. For head-and-neck cancer, the margins were adjusted to 1.0 cm beyond the GTV to obtain the CTV; the CTV was expanded symmetrically by 0.3 cm in all directions to account for patient setup and motion within the thermoplastic mask. For prostate cancer the CTV was considered to be the prostate plus seminal vesicles; the planning treatment volume (PTV) was obtained by expanding in 3D the CTV by 1.0 cm and 0.7 cm on the prostate–rectum interface to avoid excessive rectal wall involvement.

All patients, except for five head-and- neck cases, were treated with one fraction per day, 5 days a week, with the fraction dose equal to 2Gy at the ICRU reference point [27]. Five head-and-neck treatments receiving 69.96 Gy to PTV1 and 59.40 Gy to PTV2 with simultaneous integrated boost in 33 fractions, the remaining ones received 70.0 Gy, according to clinical risk. Respectively for lung, prostate and breast cancer the prescription dose was 60.0, 76.0 and 50.0 Gy.

The treatment plans were developed using Eclipse 8.1 TPS (Treatment Planning System); the dose distributions of the clinical treatment plans initially performed using the PBC algorithm were recalculated with AAA using the same number of monitor units provided by PBC.

The paired Student t-test was used for statistical comparison of all results obtained from the use of the two algorithms. All tests were two-tailed with a p value of < 0.05 considered the threshold for statistical significance.

For the validation of both the algorithms implemented in the TPS, the tests, the analyses, and the acceptability criteria were in large part based on the report of the AAPM Report 55 [28], and other documents such as the technical report by IAEA [29] were consulted. For the AAA, the outcomes of some tests were comparable to those provided by Van Esch et al. [14].

### Dose analysis

For the PTV we evaluated D_95%_, D_2%_ dose levels on the dose volume histogram (DVH) above which lay 95% and 2% of the volume of the PTV; they were used as a surrogate for dose minimum and dose maximum, respectively. The mean dose to the PTV was also considered.

To describe the degree of the PTV dose inhomogeneity, the Inhomogeneity Index (II) was used and it has been calculated as (*D*_2*%*_ − *D*_95*%*_)/*D*_*median*_, II is equal to 0 if no intra-target inhomogeneity is observed.

For breast treatments, D_15%_, D_2%_ and the mean dose, D_mean_, to the heart and homolateral lung were assessed; D_2%_, D_20%_, D_60%_, V_20_ (relative volume of the lung receiving at least 20.0 Gy) and D_mean_ to the lung-CTV for NSCLC treatments were considered; mean parotid glands dose for head-and-neck treatment; finally D_2%_, D_50%_, D_95%_ and D_mean_ to the rectum and D_2%_, D_50%_, D_80%_ to femoral heads for prostate treatments were recorded.

### NTCP and TCP analysis

The NTCP were evaluated by applying two different radiobiological models: the Lyman-Kutcher-Burman (LKB) model [21-23,30] and the relative seriality (RS) model [31]. For the five head-and-neck simultaneous integrated boost treatments, the linear quadratic equivalent doses at 2 Gy per fraction (EQD_2_) DVHs were calculated; α∕β = 3 Gy was assumed.

The NTCP and TCP calculation was performed by a home-made software; the dose calculation in Eclipse was calculated using the minimum available grid size, 0.25 cm, and the step size for differential DVH export was chosen to be 25 cGy, it was a compromise between time necessary for TCP/NTCP calculation and the accuracy of the computed values.

Six different combinations of NTCP model/parameters were available for the heart, three quantifying the risk for pericarditis, and three the excess risk for cardiac mortality. For the lung we applied eight different model parameter sets relating to the development of pneumonitis. The lung-GTV is the volume considered from some of the used NTCP parameter sets (e.g. De Jaeger et al. [24]) and using these parameters on the lung-CTV volume would generally be incorrect, as any differences in dose distribution from the PBC or AAA is most noticeable at the lung/tumour interface. However because the tumor was at an advanced stage and the GTV was similar to the CTV, lung-GTV and lung-CTV were only slightly different. Therefore using the NTCP model parameters for lung-GTV on the lung-CTV may be suitable.

For quantifying the risk of xerostomia from irradiation of the parotid glands we used three different combinations of NTCP model/parameters. Seven sets of parameters were used for the prediction of late rectal bleeding in the patients treated for prostate cancer and only one set of parameters relating to the necrosis of femoral heads. The applied parameters are listed in Tables 1, 2, 3, 4, and 5.

**Table 1:** Summary of NTCP modeling studies of cardiac toxicity

**Organ: Whole heart/ Pericard; End**-**point: Pericarditis**				
**Ref.**	**LKB model parameters**			**Notes**
	**n**	**D**_**50**_**(Gy)**	**m**	
Emami et al. [39] / Burman et al. [23]^†^	0.35	48.00	0.10	whole heart
Martel et al. [57]	0.636	50.60	0.13	pericard (1cm thick shell)
**Ref.**	**Seriality model parameters**			**Notes**
	**γ**	**D**_**50**_**(Gy)**	**s**	
Emami et al. [39] / Ågren-Cronqvist [40]^‡^	3	49.20	0.2	whole heart
Organ: Whole heart; End-point: Excess card.mortality				
Gagliardi et al. [58]	1.28	52.40	1.00	
Eriksson et al. [59]	0.93	63.30	1.00	
Eriksson et al. [59]	0.96	70.30	1.00	

^†^Clinical data from Emami et al. [39], model fitting by Burman et al. [23].

^‡^Clinical data from Emami et al. [39], model fitting by Ågren-Cronqvist [40].

**Table 2:** Summary of NTCP modeling studies of lung toxicity

**Organ: Lung; End**-**point: Pneumonitis**				
**Ref.**	**LKB model parameters**			**Notes**
	**n**	**D**_**50**_**(Gy)**	**m**	
Emami et al. [39] / Burman et al. [23]^†^	0.87	24.50	0.18	
Kwa et al. [60]	1.00	30.50	0.30	
Seppenwoolde et al. [38]	0.99	30.80	0.37	
De Jeager et al. [24]^*^	1.00	34.10	0.45	
De Jeager et al. [24]^**^	1.00	29.20	0.45	
**Ref.**	**Seriality model parameters**			**Notes**
	**γ**	**D**_**50**_**(Gy)**	**s**	
Emami et al. [39] / Ågren-Cronqvist [40]^‡^	2.10	24.50	0.0061	
Seppenwoolde et al. [38]	0.900	34.00	0.060	
Gagliardi et al. [61]	0.966	30.10	0.012	lungs were considered as separate organs

^†^Clinical data from Emami et al. [39], model fitting by Burman et al. [23].

^*^ Tissue inhomogeneity correction: Equivalent-path.

^**^ Tissue inhomogeneity correction: Convolution-superposition.

^‡^Clinical data from Emami et al. [39], model fitting by Ågren-Cronqvist [40].

**Table 3:** Summary of NTCP modeling studies of parotid glands toxicity

**Organ: parotid glands; End**-**point**: **Total Xerostomia / 25% Xerostomia**				
**Ref.**	**LKB model parameters**			**Notes**
	**n**	**D**_**50**_**(Gy)**	**m**	
Emami et al. [39] / Burman et al. [23]^†^	0.70	46.00	0.18	total xerostomia
Eisbruch et al. [41]	1.00	28.40	0.18	25% xerostomia at 1 year
Roesink et al. [42]	1.00	39.00	0.45	25% xerostomia at 1 year

^†^Clinical data from Emami et al. [39], model fitting by Burman et al. [23].

**Table 4:** Summary of NTCP modeling studies of rectal toxicity

**Organ: Rectum; End-point: Late rectal bleeding**				
**Ref.**	**LKB model parameters**			**Notes**
	**n**	**D**_**50**_**(Gy)**	**m**	
Rancati et al. [62]	0.23	81.90	0.19	solid rectum including filling
Rancati et al. [62]	0.06	78.60	0.06	solid rectum including filling
Tucker et al. [45]	0.08	78.00	0.14	solid rectum including filling
Peeters et al. [63]	0.13	80.70	0.14	rectal wall
Söhn et al. [47]	0.08	78.40	0.11	rectal wall
Rancati et al. [46]	0.085	97.70	0.27	solid rectum including filling
**Ref.**	**Seriality model parameters**			**Notes**
	**γ**	**D**_**50**_**(Gy)**	**s**	
Rancati et al. [62]	1.69	83.10	0.49	solid rectum including filling

**Table 5:** NTCP modeling study of femoral heads toxicity

**Organ: Femoral heads; End**-**point: Necrosis**				
**Ref.**	**LKB model parameters**			**Notes**
	**n**	**D**_**50**_**(Gy)**	**m**	
Emami et al. [39] / Burman et al. [23]^†^	0.25	65.00	0.12	

^†^Clinical data from Emami et al. [39], model fitting by Burman et al. [23].

Using the LQ model the TCP was also calculated from the DVH of the CTV. For head-and-neck, breast and lung tumor α/β ratio of 10 Gy was used. At present much debate is going on about the value of α/β for prostate cancer. Modeling studies suggest that α/β could be as low as 1.49 Gy, while other studies show higher α/β [32-37]. Because valid data were not available, we decided to do our investigation with two extreme values; a α/β ratio of 1.49 Gy [32] and 10 Gy [37] were chosen, subsequently, these sets of parameters are referred to as α/β_1.49 and α/β_10 respectively.

## Results

The results of the comparison of the treatment plans as calculated by two algorithms, PBC and AAA, are summarized in Tables 6, 7, 8, and 9 for breast, lung, head-and-neck and prostate treatment respectively.

**Table 6:** Summary of differences between treatment plan and radiobiological parameters from the two algorithms for breast treatment

	**Parameters**		**AAA Mean**	**sd**	**PBC Mean**	**sd**	**p**
PTV	D_2%_(Gy)		51.5	0.7	52.2	0.7	<0.001
	D_95%_(Gy)		47.1	0.8	48.7	0.6	<0.001
	D_mean_(Gy)		49.2	0.7	50.4	0.6	<0.001
	II (%)		8.9	0.4	6.9	0.3	<0.001
	TCP(%)		77.3	7.7	85.1	4.3	<0.001
Left Lung	D_2%_(Gy)		44.1	1.3	48.0	1.6	<0.001
	D_15%_(Gy)		7.0	1.5	4.0	1.2	<0.001
	D_mean_(Gy)		5.9	1.2	4.1	1.2	<0.001
		LKB model					
	NTCP (%)	Ref.					
		Emami et al. [39] / Burman et al. [23]	0.0	0.0	0.0	0.0	
		Kwa et al. [60]	0.2	0.1	0.1	0.0	<0.008
		Seppenwoolde et al. [38]	0.7	0.1	0.6	0.1	<0.005
		De Jeager et al. [24]^†^	2.0	0.3	1.6	0.2	<0.001
		De Jeager et al. [24]^††^	2.1	0.3	1.8	0.2	<0.001
		Seriality model					
		Emami et al. [39]/ Ågren-Cronqvist et al. [40]	0.0	0.0	0.0	0.0	
		Seppenwoolde et al. [38]	0.2	0.1	0.1	0.1	<0.001
		Gagliardi et al. [61]	0.2	0.1	0.1	0.1	0.020
Heart	D_2%_(Gy)		37.7	12.5	37.5	13.3	**0.758**
	D_15%_(Gy)		3.9	0.7	2.8	0.6	<0.001
		LKB model					
	NTCP(%)	Ref.					
		Emami et al. [39] /Burman et al. [23]	0.0	0.0	0.0	0.0	
		Martel et al. [57]	0.0	0.0	0.0	0.0	
		Seriality model					
		Emami et al. [39]/Ågren-Cronqvist et al. [40]	0.0	0.0	0.0	0.0	
		Gagliardi et al. [58]	0.6	0.4	0.6	0.4	**0.243**
		Eriksson et al. [59]	0.6	0.3	0.6	0.3	**0.482**
		Eriksson et al. [59]	0.4	0.2	0.4	0.2	**0.418**

^†^ Tissue inhomogeneity correction: Equivalent-path.

^††^ Tissue inhomogeneity correction: Convolution-superposition.

Insignificant differences (p > 0.05) are in bold.

**Table 7:** Summary of differences between treatment plan and radiobiological parameters from the two algorithms for lung treatment

	**Parameters**		**AAA Mean**	**sd**	**PBC Mean**	**sd**	**p**
PTV	D_2%_(Gy)		61.4	0.3	63.2	0.3	<0.001
	D_95%_(Gy)		56.0	0.4	57.9	0.5	<0.001
	D_mean_(Gy)		58.7	0.4	60.5	0.3	<0.001
	II(%)		11.0	0.6	9.0	0.6	<0.001
	TCP(%)		78.4	3.0	86.7	1.0	<0.001
Lung	D_2%_(Gy)		55.7	4.8	59.3	5.5	<0.001
	D_20%_(Gy)		23.4	9.7	23.2	10.9	**0.880**
	D_60%_(Gy)		4.1	3.0	3.0	2.5	<0.001
	D_mean_(Gy)		17.5	5.0	17.1	5.1	**0.281**
	V_20_(%)		26.2	12.9	24.5	13.2	0.045
		LKB model					
	NTCP (%)	Ref.					
		Emami et al. [39] /Burman et al. [23]	28.5	16.3	33.0	16.3	0.040
		Kwa et al. [60]	7.0	4.9	8.0	5.9	0.026
		Seppenwoolde et al. [38]	8.9	5.4	9.8	6.1	<0.001
		De Jeager et al. [24]^†^	10.6	4.8	11.4	5.2	0.024
		De Jeager et al. [24]^††^	14.9	7.2	15.6	7.0	**0.055**
		Seriality model					
		Emami et al. [39] /Ågren-Cronqvist [40]	23.7	15.7	27.6	15.8	0.022
		Seppenwoolde et al. [38]	7.9	4.7	8.8	5.4	<0.001
		Gagliardi et al. [61]^*^	18.8	8.0	21.3	8.9	<0.001
		Gagliardi et al. [61]^**^	14.4	9.1	15.9	9.9	<0.001

* left lung; ** right lung.

^†^ Tissue inhomogeneity correction: Equivalent-path.

^††^ Tissue inhomogeneity correction: Convolution-superposition.

Insignificant differences (p > 0.05) are in bold.

**Table 8:** **Summary of differences between treatment plan and radiobiological parameters from the two algorithms for head**-**and**-**neck treatment**

	**Parameters**		**AAA Mean**	**sd**	**PBC Mean**	**sd**	**p**
PTV	D_2%_(Gy)		64.4	4.6	66.3	4.9	<0.001
	D_95%_(Gy)		60.2	4.6	62.5	5.0	<0.001
	D_mean_(Gy)		62.1	4.5	64.3	4.9	<0.001
	II(%)		7.0	0.3	6.0	0.4	<0.001
	TCP(%)		84.4	5.1	88.4	2.5	<0.001
parotid glands	D_mean_(Gy)^*^		35.7	1.8	36.6	1.7	<0.001
	D_mean_(Gy)^**^		34.0	4.7	34.9	4.3	0.02
	D_mean_(Gy)		35.0	4.0	36.2	3.9	<0.001
		LKB model					
	NTCP (%)	Ref.					
		Emami et al. [39] / Burman et al. [23]	12.8	3.0	15.2	2.7	0.002
		Roesink et al. [42]	33.5	2.6	36.0	2.1	<0.001
		Eisbruch et al. [41]	57.5	4.0	63.8	3.8	<0.001

^*^ left parotid gland^; **^ right parotid gland.

**Table 9:** Summary of differences between treatment plan and radiobiological parameters from the two algorithms for prostate treatment

	**Parameters**		**AAA Mean**	**sd**	**PBC Mean**	**sd**	**p**
PTV	D_2%_(Gy)		77.6	1.6	78.7	1.6	<0.001
	D_95%_(Gy)		75.3	1.6	76.2	1.5	<0.001
	D_mean_(Gy)		76.5	1.5	77.4	1.6	<0.001
	II(%)		3.2	0.1	3.2	0.2	**0.416**
	TCP(%)_α/β 1.49_		81.9	5.1	83.9	4.3	<0.001
	TCP(%)_α/β 10.0_		93.8	6.5	95.7	5.0	<0.001
Rectum	D_2%_(Gy)		74.3	1.6	77.0	2.0	<0.001
	D_50%_(Gy)		41.7	6.5	40.7	6.8	<0.001
	D_95%_(Gy)		21.1	4.8	18.6	4.7	<0.001
	D_mean_(Gy)		44.6	6.2	43.9	6.6	0.002
		LKB model					
	NTCP (%)	Ref.					
		Rancati et al. [62]	3.3	1.9	3.9	2.5	0.005
		Rancati et al. [62]	0.5	0.6	1.5	2.0	0.006
		Tucker et al. [45]	9.2	2.2	11.5	2.3	<0.001
		Peeters et al. [63]	3.1	1.9	4.1	2.9	<0.001
		Söhn et al. [47]	4.5	1.8	6.4	2.6	<0.001
		Rancati et al. [46]	9.1	2.2	11.1	2.2	<0.001
		Seriality model					
		Rancati et al. [62]	3.0	1.8	3.5	2.4	0.013
Femoral heads	D_2%_(Gy)		55.2	3.6	54.5	3.8	**0.104**
	D_50%_(Gy)		44.6	6.9	43.6	7.3	<0.001
	D_80%_(Gy)		26.1	5.7	25.1	6.0	<0.001
		LKB model					
	NTCP(%)	Ref.					
		Emami et al. [39] / Burman et al. [23]	0.2	0.1	0.1	0.1	<0.001

Insignificant differences (p > 0.05) are in bold.

Subsequently, NTCP calculated with the AAA and PBC algorithm are referred to as NTCP_AAA_ and NTCP_PBC_, respectively; the NTCP values less than 0.1% are assumed to be zero.

### The breast treatment

**Figure 1:**
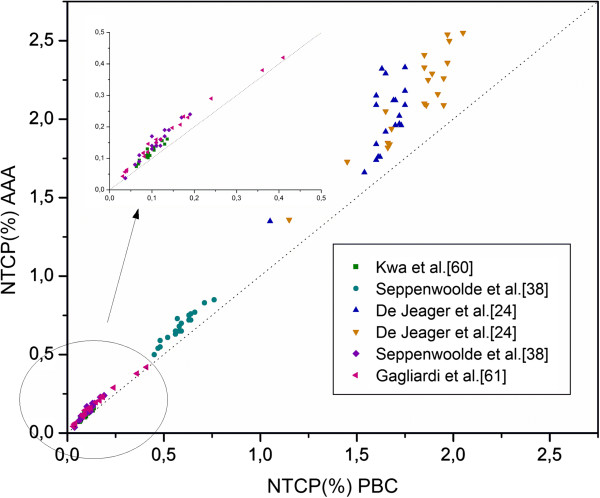
**Comparison of NTCP for risk of developing pneumonitis following breast treatment computed with the AAA** (**ordinate**) **and the PBC algorithm** (**abscissa**) **for NTCP models**/**parameters sets from Table**2. Each symbol represents data of an individual patient. The dotted line indicates the line of *identity*.

**Figure 2:**
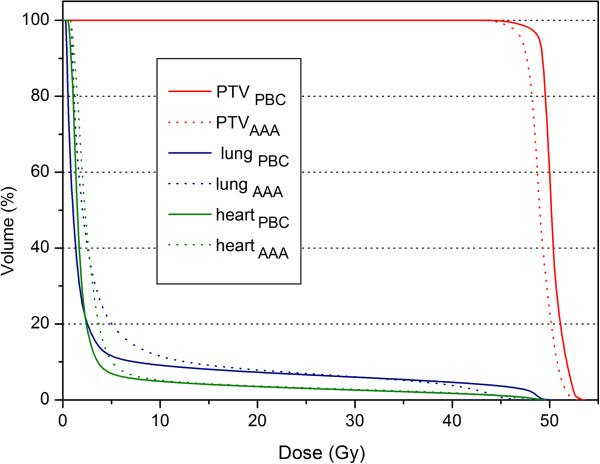
**Example of a comparative DVH for a breast plan.** The curves calculated by the PBC algorithm are depicted by solid lines and those calculated by the AAA by dotted lines.

When AAA was used, the maximum percentage difference was −3.3% for D_95%_ and a increase of 2.0% for II was found (Table 6). The poorer coverage of the PTV was reflected in the TCP, which was significantly lower when the AAA was used, the mean value was 77.3% (sd = 7.7%) and 85.1% (sd = 4.3%) for PBC (p < 0.001). For the ipsilateral lung while mean D_2%_ decreased when the AAA was applied, the mean D_15%_ and D_mean_ increased by 3.0 Gy and 1.8 Gy respectively; the mean NTCP_AAA_ values were higher than NTCP_PBC_ (Figures 1 and 2)_._ The mean NTCP_AAA_ varied from 0.2% (sd = 0.1%) to 2.1% (sd = 0.3%), while the mean NTCP_PBC_ varied from 0.1% (sd = 0.0%) to 1.8% (sd = 0.2%) depending on the chosen parameters set.

A mean increase of 0.1% was observed on the NTCP_AAA_ value when it was estimated by De Jeager et al. [24] parameters and re-evaluated using convolution-superposition (CS) algorithm for the dose calculation. When the Seriality Model was applied, the NTCP value estimated by Seppenwoolde et al. [38] was lower than had been predicted by the LKB model.

The incidence of radiation pneumonitis predicted by both Emami et al. [39] / Burman et al. [23] and Emami et al. [39] / Agren-Cronqvist [40] parameters was zero.

Following left breast cancer treatments, the risk of excess cardiac mortality was found to be low; there was no statistically significant difference between the AAA and PBC. The risk for developing pericarditis was zero for all the considered NTCP parameters. These results might be due to little cardiac tissue having been exposed to radiation beam, but on the other hand it is also necessary to consider that presently there are insufficient clinical data on the dose–response characteristics of cardiac tissue on which to base reliable estimates of radiobiological parameters.

### The lung treatment

Changes were observed specifically in the PTV coverage; there was a mean percentage difference of about −3% for the D_95%_, D_2%_ and D_mean_ when the AAA was used and as a result, a mean decrease of 8.3% for TCP was observed (Table 7).

**Figure 3:**
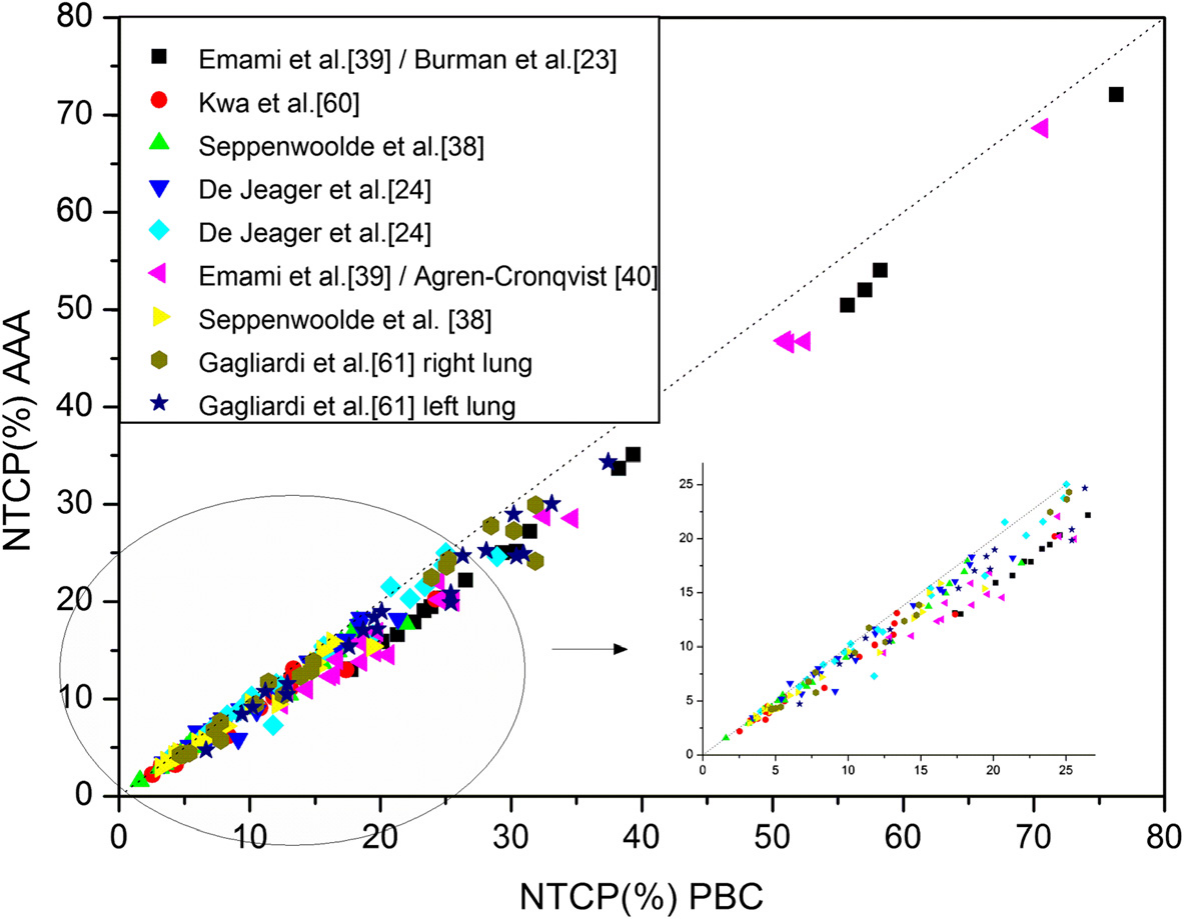
**Comparison of NTCP for risk of developing pneumonitis following NSCLC treatment computed with the AAA** (**ordinate**) **and the PBC algorithm** (**abscissa**) **for NTCP models**/**parameters sets from Table**2. Each symbol represents data of an individual patient. The dotted line indicates the line of identity.

For the normal lung dose parameters, the AAA predicted a mean reduction of 3.6 Gy for D_2%_, while the differences for D_mean_ and D_20%_ were not statistically significant. D_60%_ and V_20%_ were found to be slightly higher when calculated with the AAA than had been predicted by the PBC algorithm. The resultant NTCP_AAA_ values were lower than the NTCP_PBC_ (Figure 3). For both Seppenwoolde et al. [38] and Emami et al. [39] / Burman et al. [23] parameters, the comparison of NTCP values as calculated by two models, LKB and RS, involved higher values if the LKB model was applied.

When De Jaeger et al. [24] parameters were used we found a mean increase of 4.3%: 14.9% (sd=7.2%) versus 10.6% (sd=4.8%), if the NTCP_AAA_ was calculated with the parameters evaluated from dose distribution based on a CS algorithm.

### The head-and-neck treatment

−3.7%, -2.9% and −3.4% were the percentage difference between AAA and PBC for D_95%_, D_2%_ and D_mean_ respectively, and the differences were statistically significant (Table 8). A reduction of 4% was observed in the mean TCP with the use of the AAA.

The mean NTCP_AAA_ of parotid gland toxicity values were lower than NTCP_PBC_. Furthermore the NTCP value obtained using Emami et al. [39]/Burman et al. [23] parameters was lower than the value predicted by the other sets of NTCP parameters. Using the Eisbruck et al. [41] parameters, the risk of a decrease in the salivary flow to 25% of the pre-treatment flow at 1 year post treatment was much higher (see Table 8) than the risk calculated by Roesink et al. [42] parameters which considered the same endpoint.

### The prostate treatment

**Figure 4:**
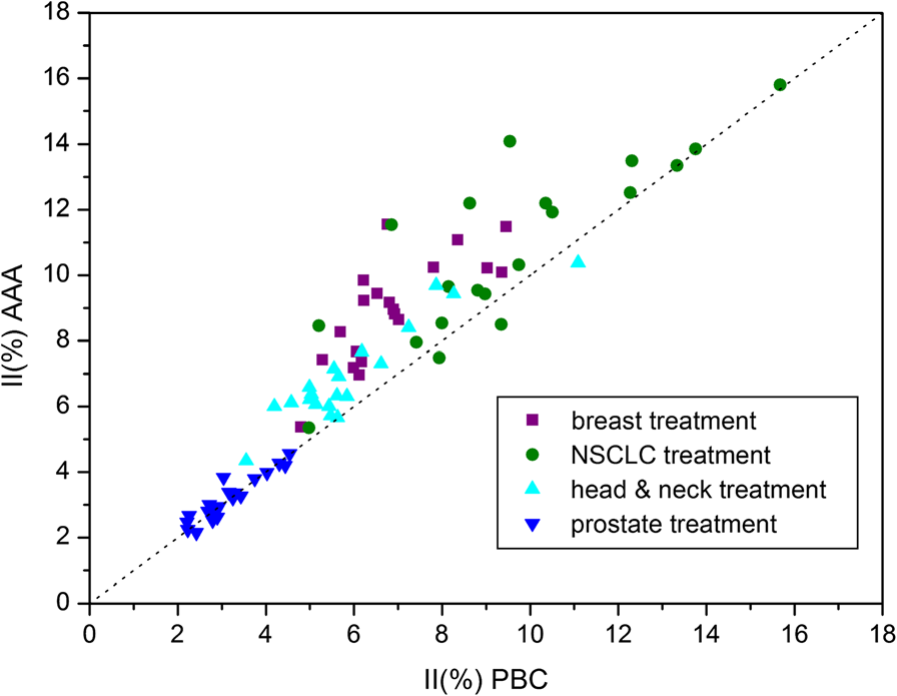
**Comparison of inhomogeneity index for treatment plans computed with the AAA** (**ordinate**) **and the PBC algorithm** (**abscissa**) **for the breast**, **NSCLC**, **head**-**and**-**neck and prostate treatments.** Each symbol represents data of an individual patient. The dotted line indicates the line of identity.

**Figure 5:**
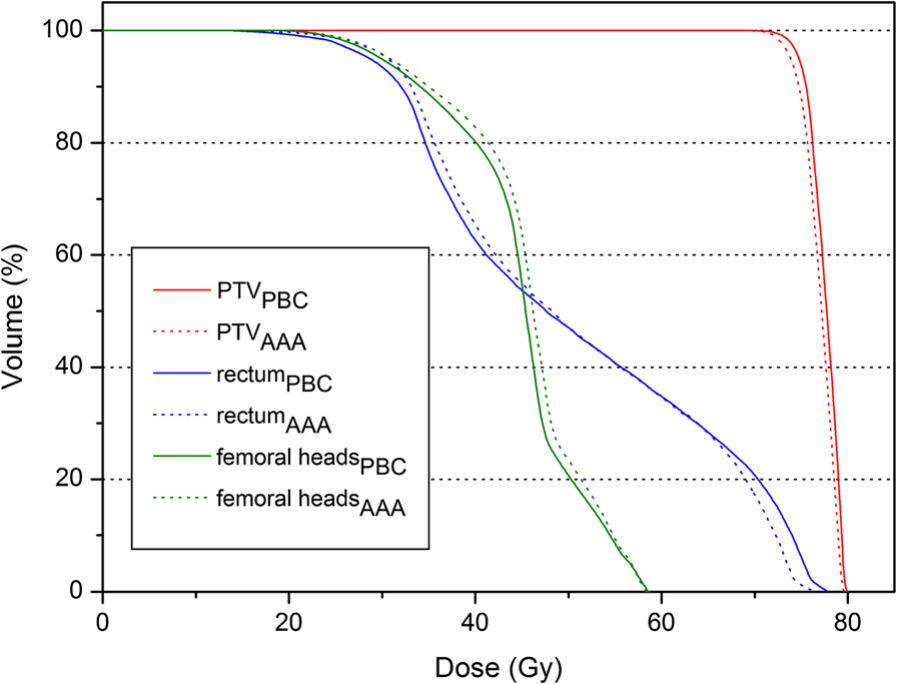
**Example of a comparative DVH for a prostate plan.** The curves calculated by the PBC algorithm are depicted by solid lines and those calculated by the AAA by dotted lines.

This type of treatment showed a markedly different pattern. The present study showed no clinically significant differences for any of the evaluated PTV dose parameters (Table 9). The type of algorithm did not affect the II (Figure 4). Across the two algorithms, the TCP was within 2.0%. Regarding the rectum, the AAA predicted slightly higher values for the dose parameters (though statistically significant) except D_2%_. But on the other hand when the PBC was used, low percentage of rectum volume was exposed to a higher dose than had been obtained with the AAA and the NTCP_PBC_ was higher than NTCP_AAA_ (Figure 5).

The tissue-bone interfaces were encountered by the lateral fields (90°, 270°); the slight shift towards higher doses of the femoral heads DVH curve was found when the AAA was used (Figure 5). Consequently, the only available combination of parameters (Emami et al. [39] / Burman et al. [23]) predicted a slightly higher risk of necrosis, even if of limited clinical significance.

## Discussion

Since the AAA is considered to be a more accurate dose computation algorithm, the comparison between the AAA and PBC dose distributions by the analyzed dose indices, provides an indication of the difference between the dose predicted by the PBC and that actually delivered. In our study the consequences of the difference on the dose-effect relations for normal tissue injury were analyzed, comparing different NTCP model/parameters.

The influence of the low density lung in proximity to the target volume was the factor largely responsible for the differences between the algorithms’ dose distributions for the breast plans. When AAA was used, the increase of II was the result of the broadening of the penumbra which was nicely modeled by the AAA; the TCP was significantly lower.

Using a more accurate algorithm the radiation in the lung was clearly transported further away; when the AAA was used the mean D_2%_ to the lung decreased by 8.1%. This is in accordance with the findings by Knöös et al. [16], which in their comparison of dose calculation algorithms divided the algorithms in two groups based on how changes in electron transport were accounted for; for the breast plans, they found that the D_5%_ to the adjacent lung decreased by 9.5% or more when accurate algorithms were used. While mean D_2%_ decreased when the AAA was applied, the mean D_15%_ and D_mean_ increased; as result, the mean NTCP_AAA_ values were higher because the available NTCP parameter sets described the lung by a prevalent parallel architecture for the analyzed endpoint. The incidence of radiation pneumonitis predicted by both Emami et al. [39] / Burman et al. [23] and Emami et al. [39] / Agren-Cronqvist [40] parameters was zero. It is worth remembering that the Emami et al. [39] study was derived from the clinical experience of the 2D planning era, without any individually assessed dose-volume information; that most external radiation therapy was delivered with opposing fields and the normal tissue was irradiated with a fairly uniform dose fraction size.

It would be interesting to know the NTCP values for the parameters estimated from Emami’s study. However more reliable parameterizations, fitting radiobiological models to clinical and dosimetric data, have been published and have to be considered to predict the risk of normal tissue toxicity.

In the lung treatment, the extremely inhomogeneous lung region resulted in the major difference of the two algorithms’ abilities to account for inhomogeneities on the final dose distribution. The factor responsible was the widening of the penumbra, in fact looking closer at the isodoses in proximity to the target revealed that they had a larger separation when the more accurate AAA was applied and was worse for those treatments where a large amount of lung tissue is involved in the PTV. These results are in agreement with the report of Bragg et al. [13], who found that lung plans generated the most significant issues in PTV coverage. When the AAA was used, a reduction for D_2%_ was observed, while the differences for D_mean_ and D_20%_ were not statistically significant, contrary to what was observed in the breast plans. The NTCP_AAA_ values were lower than the NTCP_PBC_. The highest NTCP values and the maximum differences between NTCP_AAA_ and NTCP_PBC_ were found for Emami et al. [39] parameters. One can see from Table 2 how much these parameters are markedly different than the other parameterizations, whether for the LKB model or for the Seriality model.

The results obtained show how the use of NTCP parameters based on more accurate dose calculation should be recommended to avoid underestimating the calculated values of NTCP. When De Jaeger et al. [24] parameters were used the underestimate was not significant when the lung tissue volume involved was small, such as for breast treatments, but it was relevant when the volume was major such as for lung treatments; we found a mean increase of 4.3%, when the NTCP_AAA_ was calculated with the parameters evaluated from dose distribution based on a CS algorithm.

In the head-and-neck treatments, as every beam passed through a region of low density material the resulting dose distributions of the two algorithms were dissimilar. A reduction of the mean TCP and of the mean NTCP values, quantifying parotid gland toxicity, was observed with the use of the AAA.

Moreover using the Eisbruck et al. [41] parameters, the NTCP value was much higher than the risk calculated by Roesink et al. [42] parameters which considered the same endpoint. This can be taken as a warning to radiation oncologists: before introducing a predictive model into clinical practice, it is necessary to assess if its predictions “make sense” in regard to that clinic’s treatment plans and experience [43,44].

The dose distribution across the two algorithms was found to be very similar in the prostate plans; no clinically significant differences for any of the evaluated PTV dose parameters were observed. Such small differences between the dose distributions were found because only two of the five beams used for these treatments were such that there was involvement of heterogeneities.

When the PBC was used, a low percentage of rectum volume was exposed to a higher dose than had been obtained with the AAA and the NTCP_PBC_ was higher than NTCP_AAA_. That is consistent with a prevalent serial architecture of the rectum for the analyzed endpoint. The effect of the variability of the NTCP parameters on the NTCP value is shown in Table 9. The major differences between the two algorithms were for those set of parameters with the n-parameter closer to 0; the highest NTCP values were with the parameters proposed by Tucker et al. [45] and Rancati et al. [46]. It is interesting to note how a slight difference for the m-value and D_50_-value between Tucker et al. [45] and Söhn et al. [47] parameters resulted in an important difference in the corresponding NTCP values.

The comparison of the two algorithms in the present study is in accordance with the literature; the differences are of minor clinical significance in many situations such as for prostate treatments and probably for other lesions in the pelvic area. The adoption of the AAA into clinical treatment planning practice requires one to fully understand its effect and its potential consequences so as to re-evaluate an assessment of dose-effect relationships and of parameters used in treatment planning decisions [48]. Similarly the introduction of a predictive model into clinical practice has to be prudent as it is necessary to assess if it is based on calculations and treatments similar to those for which the NTCP has to be calculated. The results found in this study show how the NTCP is strongly affected by the wide-ranging values of radiobiological parameters and the differences between the dose distributions of the two tested algorithms yield statistically significant differences in the NTCP values.

## Conclusions

In this study, we have tried to investigate qualitative, possible clinical consequences of the use of PBC versus AAA (keeping the same number of monitor units provided by PBC and clinically delivered to each patient) by comparing different NTCP model/parameters. As general result, the NTCP_AAA_ was lower than the NTCP_PBC_, except for the breast treatments.

The difference in NTCP between PBC and AAA treatment plans could be clinically significant and it may be the subject of a future prospective study.

Moreover we have observed how much the NTCP value depends strongly on the choice of radiobiological parameters. Radiobiological modeling can play an important role in high quality radiotherapy, however uncritical reliance on model results may compromise treatment outcomes and patient safety. It is important to use NTCP parameter sets based on calculations and treatments similar to those for which the NTCP has to be calculated; additionally, it is necessary to improve models and obtain more robust radiobiological parameters [49-56].

## Abbreviations

AAA: Anisotropic analytical algorithm; DVH: Dose volume histogram; CC: Collapsed cone; CS: Convolution-superposition; CTVs: Clinical target volumes; Dx%: Dose level on the; DVH: above which lay x% of the observed volume; 3DCRT: Three-dimensional conformal radition therapy; GTV: Gross tumor volume; IMRT: Intensity modulated radiation therapy; LKB: Lyman-kutcher-burman model; MC: Monte Carlo; NSCLC: Non-small cell lung cancer; NTCP: Normal tissue complication probability; OARs: Organs at risk; PBC: Pencil beam convolution algorithm; PTV: Planning treatment volume; RS: Relative seriality model; TPS: Treatment planning system.

## Competing interests

The authors declare they have no competing interests.

## Authors’ contributions

AB designed the study, performed the analyses and wrote the manuscript; BN carried out the data from DVH of the treatment plans and helped to perform the analyses; RC helped to make graphs and tables; LB read the different manuscript’s versions. All authors read and approved the final manuscript.
